# (Epi)genetic Inheritance in *Schistosoma mansoni:* A Systems Approach

**DOI:** 10.1016/j.pt.2016.12.002

**Published:** 2017-04

**Authors:** Céline Cosseau, Olaf Wolkenhauer, Gilda Padalino, Kathrin K. Geyer, Karl F. Hoffmann, Christoph Grunau

**Affiliations:** 1 University Perpignan Via Domitia, IHPE UMR 5244, CNRS, IFREMER, University Montpellier, F-66860 Perpignan, France; 2 Dept of Systems Biology & Bioinformatics, University of Rostock, D-18051 Rostock, Germany; 3 Stellenbosch Institute for Advanced Study (STIAS), Wallenberg Research Centre, Stellenbosch, South Africa; 4 Institute of Biological, Environmental and Rural Sciences (IBERS), Edward Llwyd Building, Room 3-31, Aberystwyth University, Ceredigion, SY23 3DA, UK

## Abstract

The *G*×*E* concept, in which genotype × environment interactions bring about the phenotype, is widely used to describe biological phenomena. We propose to extend the initial notion of the concept, replacing *G* by ‘inheritance system’. This system, comprised of both genome and epigenome components, collectively interacts with the environment to shape the development of a phenotype. In the case of the human blood fluke *Schistosoma mansoni*, responsible for intestinal bilharzia, the phenotypic trait that is most relevant to global health is infection success. Taking a systems biology view we show how genetic and epigenetic interactions result in ephemeral, but also heritable, phenotypic variations that are important for infection success.

## Trends

There is an intense debate about the importance of nongenetic inheritance for evolution.

*Schistosoma mansoni* epigenotype components are important players in parasite development and host interactions.

We use *S. mansoni* as an example to introduce the notion of ‘inheritance system’ in which genotype and the epigenotype components collectively interact with the environment to shape the development of a phenotype.

## The Many Facets of Heritable Phenotypic Diversity in Schistosomes

**Figure 1:**
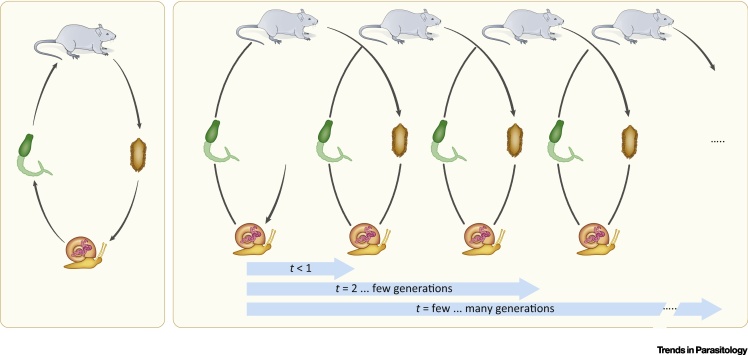
Schematic Representation of the Life Cycle of *Schistosoma Mansoni* in the Context of a Systems Approach to Inheritance. On the left, a classical view of the life cycle of *S. mansoni*. Morphologically different male and female adults mate in the definitive mammalian host, produce embryonated eggs that release miraciadia when in contact with fresh water. Miracidia seek and penetrate *Biomphalaria* snails, transform into asexually reproductive sporocysts and eventually develop into cercariae. These larvae infect the mammalian host and develop, during a complex migration in the host body via schistosomula, into adult worms. This schematic representation of the life cycle is reduced and does not include a time component, thereby losing its nature as a process. On the right, the same life cycle representation but with time as vector from left to right, that is, showing its process characteristics. Even if an identical individual could serve as intermediate- (snail) or definitive- (rodent or human) host, this individual is not the same as when it experienced the first infection.

*S. mansoni*, the agent of human intestinal bilharzia, causes an estimated annual loss of 3–15 million disability-adjusted life-years 1, 2. This digenean flatworm has a complex life cycle with human and primates as natural definitive hosts and freshwater snails as intermediate hosts. Small rodents can also be infected and enable maintenance of the life cycle in the laboratory (Figure 1). *S. mansoni* originates from the East African lake area and managed to invade the South American subcontinent during the 15th to 19th centuries via the slave trade [3]. In this new environment it uses an intermediate host snail species that had segregated from its African snail host several million years earlier [4]. In this review we intend to summarize some of the principles of the capacity of *S. mansoni* to generate sufficient heritable phenotypic diversity to explain this long and very successful history in adapting to new hosts. Nevertheless, we believe that many of the principles we will outline can be applied to the African and Asian *Schistosoma* species. The need for a better understanding of *S. mansoni* sister lineages, such as *Schistosoma bovis* and *Schistosoma haematobium*, becomes urgent even in nontropical regions given the recent infection of human hosts by a hybrid of both in the European continent [5].

Humans are part of this story, and understanding our common past might enable us to shape our future common history in a way that allows us to manage the disease. Heritable phenotypic diversity has traditionally been attributed to genetic diversity. We know today that this view is not wrong but must be complemented by other diversity-generating mechanisms that can be heritable, such as those regulated by epigenetic elements or symbiotic organisms. It is, for instance, well known that pathogenicity of *Onchocerca volvulus*, the agent of river blindness, depends on infection with the obligatory intracellular bacterium *Wolbachia* [6], and that the capacity of parasitoids (e.g., the wasp *Dinocampus coccinellae*) to modulate insect host behavior is based on symbiotic viruses [7]. Despite very clear examples of nongenetic-based inheritance, DNA is still seen as the major inheritance system, with other components playing accessory roles. Some controversy around this topic has arisen with considerable scientific debate about the importance of nongenetic and, in particular, epigenetic inheritance for modulating phenotype [8]. The origin of heritable phenotypic variants has been a central question in evolutionary biology since Darwinian times. Advances in plant breeding and the generation of pure lines through self-pollination in the beginning of the 20th century led to the idea of separating the ‘visible’ nature of an organism away from its inheritance system and also to the development of the genotype (G) – phenotype (P) concept [9]. A consequence of this concept is the idea that natural selection acts on the phenotype and not on the genotype. This concept facilitated rapid progress in animal and plant breeding programs but also supported the view that life history traits depend not only on the pedigree but also on the conditions in which the offspring was raised.

In the late 1940s, Haldane and others [10] extended the G–P model into what is today known as the *G*×*E* concept, meaning that genotype × environment interactions bring about the phenotype [11]. It should be noted that, up until the 1950s, ‘gene’ was defined as a ‘unit’ or ‘element’, and inheritance was implicit since ‘genes’ were contained within gametes [9]. With advancements in molecular biology, this notion changed, and today ‘genes’ are considered by most as DNA fragments that code for proteins [12]. The epigenotype definition followed the same logic: when the term was coined by Waddington, it was related to epigenesis, that is, how genotypes give rise to phenotypes during development [13]. Only recently, however, has epigenetics been redefined as “the study of mitotically and/or meiotically heritable changes in gene function that cannot be explained by changes in DNA sequence” [14]. Sometimes, the DNA is compared to the hardware of a computer and the epigenetic information to the software, that is, a set of instructions on how to use this hardware (soft inheritance) [15]. We believe that this metaphor is not entirely wrong but might be misleading; we propose to revisit the initial notion of *G*×*E* and to extend it by borrowing ideas used within systems biology and by incorporating recent advancements in evolutionary biology 16, 17. If one returns to the original, heritability-based genotype definition, *G* can be replaced by ‘inheritance system’. This inheritance system would then be composed of several elements: the genotype *G*, the epigenotype *I*, heritable cytoplasmic elements (e.g., mitochondria and endosymbionts such as *Wolbachia* spp*.*), and symbionts (e.g., maternal microbiota). For the purpose of this review, we will focus on the dual inheritance system (*G*×*I*) that interacts with the environment *E* to bring about the phenotype *P* (conceptualized as (*G*×*I*)×*E* ⇒*P*). We will now define operationally the elements of this system (*G* and *I*) using their molecular nature, in the case of *S. mansoni* DNA for *G*, and chromatin proteins for *I*. What is important here is the interaction of the elements in the system: DNA must act on chromatin that provides feedback to DNA and *vice versa*. (*G*×*I*)×*E* is a system, but the production of the phenotype is a process. An inherent property of a process is the time *t* it takes to produce its results. Time can be measured in physical units, but in an ecological and evolutionary context it might be more useful to measure it in meiotic generations. This way, the length of the process will be defined as follows: when referring to ontogenesis, *t* < 1; parental effects, *t* = 1; transgenerational effects, tentatively defined with *t* = 3–10; and phylogeny for hundreds to thousands of meiotic generations (Figure 1). We now define the three elements of the system, that is, the genome, epigenome, and the environment, and subsequently explore how they interact to shape the phenotype of *S. mansoni* in different time scales. While we shall not formulate our discussion mathematically, our arguments and definitions related to ‘processes’, ‘system’ and ‘state’ follow Mesarovic and Takahara [18].

## Element 1: The Genome (*G*)

The genome of *S. mansoni*, and all African schistosomes, is organized into seven autosome pairs and two sex chromosomes (ZZ male, ZW female). Its total length is roughly 363 megabases of DNA, spanning about 11 000 protein-coding genes 19, 20. Half of the genome is comprised of repetitive sequences that are predominantly composed of both interspersed and satellite types [21]. Many of these repetitive elements are transcribed and could be of functional importance for the phenotype. For example, their potential impact is well illustrated by the peculiar nature of the *S. mansoni* sex chromosomes. Currently, no W-specific genes have been identified, suggesting that both males (ZZ) and females (ZW) contain at least one copy of every gene. However, there are large blocks of female-specific satellite repeats on the W chromosome [22]. Their role is difficult to assess, but it might be that they influence chromatin structure, either in *cis* or *trans*, and operate via the epigenetic element of the inheritance system (see more below).

In endemic areas, genetic diversity between *S. mansoni* populations is considered relatively high [23]. However, when isolates are brought into the laboratory, genetic diversity decreases rapidly [24]. Diversity is even lower and can be considered quasi-clonal on the level of unique sequences within these inbred laboratory populations [25]. However, an important source of genetic diversity – that has not been captured sufficiently before the use of massive sequencing – is copy number variations (CNVs). For instance, there are about 2000 CNVs between two South American isolates of *S. mansoni* that had been maintained in the laboratory for about 30–40 years [26]. In addition, we have also shown that up to about 100 CNVs can occur between experimentally produced somaclonal lines [25]. Nevertheless, it is currently unknown the extent by which CNVs influence phenotypic diversity or, indeed, the schistosome inheritance system.

## Element 2: The Epigenome (*I*)

For the purpose of this review, we define the chromatin structure as the epigenetic element of the *S. mansoni* inheritance system. Chromatin is a complex of DNA and associated proteins. Histones, the main protein component of chromatin, play an important role in DNA packaging but also carry epigenetic information. Other proteins are likely as important as histones for maintaining the chromatin structure, but very little is known about them in schistosomes. We will therefore focus on histones and their post-translational modifications (PTM). The fundamental chromatin unit, a nucleosome, consists of two stabilized histone 3-histone 4 (H3-H4) dimers flanked by two histone histone 2A-histone 2B (H2A-H2B) dimers, as well as 147 bp of DNA wound around the complete octamer 27, 28. Each of the core histones contains an N-terminal 20–30 amino acid projection called the histone tail, with amino acids in these tails often covalently modified by PTMs. More than 60 histone PTMs have been described in eukaryotes, such as acetylation, methylation, phosphorylation, ubiquitinylation, citrullination, and sumoylation. Histone modifications can regulate transcription [29] but correlative data indicate that they are probably implicated in many post-transcriptional processes, including (alternative) splicing [30].

**Figure 2:**
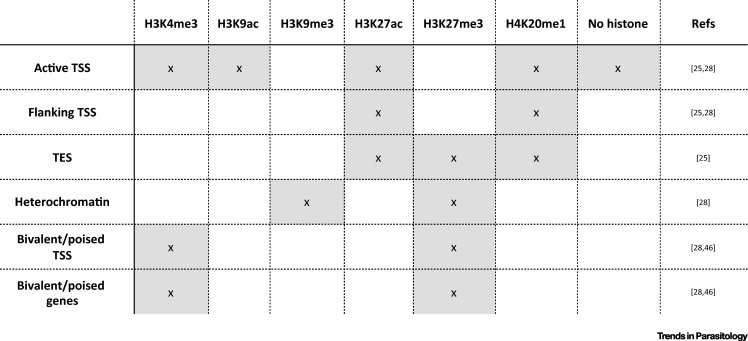
Chromatin Colors in *Schistosoma mansoni*. The classical euchromatin/heterochromatin concept was defined based on the way the compounds of the nucleoplasm could be stained with hematoxylin [61]. The terms were actually defined provisionally but subsequently frequently used to describe an open, transcriptional competent form of the chromatin (euchromatin) and the repressive, closed form (heterochromatin). Very recently, a new concept has emerged using the idea of several states (or ‘colors’) of chromatin that are defined by different combinations of histone modifications, nonhistone chromatin proteins, and DNA methylation 62, 63, 64. This figure summarizes the current knowledge on combinations of histone modifications that ‘stain’ the chromatin of *S. mansoni* (grey – over representation, white – underrepresentation) 25, 28, 46. Abbreviations: H3K4me3, histone H3 tri-methylated at lysine 4; H3K9ac, histone H3 acetylated at lysine 9; H3K9me3, histone H3 tri-methylated at lysine 9; H3K27ac, histone H3 acetylated at lysine 27; H3K27me3, histone H3 tri-methylated at lysine 27; H4K20me1, histone H4 mono-methylated at lysine 20; TSS, transcription start site; TES, transcription end site.

Histone modifications are catalyzed by enzymatic ‘writers’ such as histone acetyl transferases (HATs) and histone methyltransferases (HMTs) [31], and are known to affect each other. For instance, methylation of H3 lysine 4 (H3K4) or phosphorylation of H3 serine 10 (H3S10) blocks methylation of H3 lysine 9 (H3K9) in HeLa cells [32]. If we consider each histone modification as an element, this interplay between different histone modifications can form distinct states of chromatin, known as chromatin colors. ChIP-Seq data revealed that *S. mansoni* has at least six chromatin colors (Figure 2). Following the Kundaje terminology [32] these chromatin colors represent (1) active transcription start sites (TSS), (2) flanking TSS, (3) transcription end sites (TES), (4) heterochromatin, (5) bivalent/poised for activation TSS, and (6) bivalent/poised for activation gene bodies with the co-occurrence of activating trimethylated H3K4 (H3K4me3) and repressive trimethylated H3 lysine 27 (H3K27me3). Kundaje *et al*. [32] used ‘absence of marks’ as a color but we find it to be a feature of active TSS (Figure 2).

Histone covalent PTMs need to be appropriately recognized and carefully regulated to ensure that chromatin remains responsive to environmental alterations. These activities are fulfilled by epigenetic ‘readers’ and ‘erasers’, respectively [33]. *S. mansoni* genome mining represents an excellent starting point for identifying these participants (as recently reviewed for histone acetylation in depth by Cabezas-Cruz *et al.* [31]) and can be used to advance this understudied area of parasite biology. For example, schistosome genome analyses (genome version 5.2) predict that histone methylation is cooperatively regulated by 25 ‘readers’, 13 ‘erasers’ and 26 ‘writers’ (Table 1). The interplay of these and all other nucleosome regulators is undoubtedly complex and carefully controlled during schistosome development, which makes them incredibly interesting targets suitable for drug discovery [31]. As an example, the *S. mansoni* genome encodes a single Jumonji-domain containing histone demethylase (Smp_137240) isoform with sequence similarity to *Homo sapiens* JMJD6, the only eraser discovered to date with sole responsibility for demethylating H3/H4 arginine residues [34]. This finding, while awaiting experimental validation, provides an exciting new epigenetic target for future schistosome drug discovery initiatives should compounds be identified that selectively (based on amino acid divergence at the substrate binding site) inhibit the schistosome and not the human homolog. In the context of a functional schistosome inheritance subsystem, the cooperation of methylation (and other PTMs) readers, writers, and erasers in chromatin remodeling remains essential for the flexible transmission of signals between environment and genome as well as to ensure that genomic imprints/modifications are reversible.

**Table 1:** *Schistosoma mansoni* proteins that regulate histone methylation^a^

Writers (26 proteins)			Readers (25 proteins)^b^			Erasers (13 proteins)		
Class^e^	Isoforms^c^	Gene Id	Class	Isoforms	Gene Id	Class	Isoforms	Gene Id
SET	1	Smp^d^_078900	Tudor	1	Smp_064830	KDM	1	Smp_150560
SET	1	Smp_070170	Tudor	1	Smp_081570	KDM	1	Smp_160810
SET	1	Smp_138030	Tudor	2	Smp_097090	KDM	1	Smp_162940
SET	1	Smp_144180	Tudor	2	Smp_150850	JMJD	1	Smp_161400
SET	1	Smp_161010	Tudor	1	Smp_175680	JMJD	1	Smp_132170
SET	1	Smp_210660	Chromo	1	Smp_027300	JMJD	1	Smp_147870
SET	1	Smp_160700	Chromo	1	Smp_041760	JMJD	1	Smp_137240
SET	1	Smp_137060	Chromo	1	Smp_079650	JMJD	1	Smp_196270
SET	1	Smp_055310	Chromo	1	Smp_078280	JMJD	1	Smp_156290
SET	1	Smp_027300	Chromo	1	Smp_130470	JMJD	1	Smp_019170
SET	1	Smp_062530	Chromo	1	Smp_144550	JMJD	1	Smp_034000
SET	1	Smp_210650	Chromo	1	Smp_149240	JMJD	1	Smp_128500^b^
SET	2	Smp_140390	Chromo	1	Smp_174840	JMJD	1	Smp_180990^b^
SET	2	Smp_150850	Chromo	2	Smp_179650			
SET	1	Smp_043580	MBT	2	Smp_006250			
SET	1	Smp_000700	MBT	1	Smp_074050			
SET	1	Smp_124950	MBT	1	Smp_159100			
SET	3	Smp_121610	PWWP	1	Smp_041750			
SET	1	Smp_149380^b^	PWWP	1	Smp_105690			
SET	1	Smp_131300^b^	PWWP	1	Smp_125340			
DOT1	1	Smp_165000	PWWP	1	Smp_137060			
PRMT	3	Smp_029240	PWWP	1	Smp_154860			
PRMT	1	Smp_211290	PWWP	1	Smp_163470			
PRMT	1	Smp_070340	PWWP	1	Smp_170390			
PRMT	1	Smp_171150	PWWP	1	Smp_125050			
PRMT	1	Smp_025550						

a Adapted from [31].

b New histone methylation epigenetic components identified in *S. mansoni* (genome assembly v5.2).

c Putative number of alternatively spliced products derived from each Smp (genome assembly v5.2).

d Smp = Schistosoma mansoni protein.

e Abbreviations: Chromo, chromatin organization modifier domain containing protein; DOT1, disruptor of telomeric silencing 1, also called Kmt4; KDM = histone lysine demethylase; JMJD, Jumonji domain-containing protein; MBT, malignant brain tumor domain-containing protein; PRMT, protein arginine methyltransferase; PWWP, proline-tryptophane-tryptophane-proline motif-containing protein; SET, SET domain-containing protein (initially characterized in Su(var)3-9, Enhancer-of-zeste and Trithorax of *Drosophila melonogaster*).

## The Environment (*E*)

The generation of an infective phenotype is a developmental and evolutionary process. A process is defined by the changes of states of a system, where a state is defined by its components, at time *t*. The notion of a system implies a conceptual boundary between the interior and exterior (environment) of a system. One can then distinguish between external variables that constitute a stimulus or perturbation to the system: the internal or state variables and the variables that are observed as a response [35]. Any other observations are parameters to the state variables. If we take the parasite as a system, food intake for instance is a stimulus, and changes in the parasite's physiology are a system's response. Between the stimulus and response, three classes of processes interact – cell signaling, gene expression, and metabolism. The systems biology approach models such processes as molecular interaction networks. Taking a cell signaling network as an example, receptor-binding ligands provide the stimulus to the subcellular network, and changes in gene expression is the response. The environment of the cell, such as osmolarity and temperature (amongst others), provides the context in which intracellular reactions take place.

With the parasite as the system, the environment interacts with this system again through temperature, pH, and osmolarity but also through biochemical and physical signals that come from the host. In a conventional systems approach, the environment would only be captured indirectly. Environmental variables are usually assumed to be constant so that their influence can be indirectly captured by parameter values that link biochemical variables. This approach is sufficient to explain, for instance, how a change in osmolarity provokes hatching of *S. mansoni* miracidia when the embryonated eggs contact freshwater. However, this approach will fail when it attempts to describe development of larvae into adult worms and to explain sex differentiation during this transition. Several studies have shown that the parasite depends on endocrine and immune signals of the mammalian host to accomplish development (reviewed in [36]). In addition to the host environment, both sexes depend on each other's microenvironment and mating status (i.e., paired versus unpaired) for the maturation of both male and female parasites [37]. A recent study has reinforced this observation of pairing-dependent transcriptome alterations and further demonstrated that about 4000 gonadal expressed genes are regulated by the presence of the opposite gender [38]. The maturation process is reversible when individuals are separated for sufficiently long time. In this case (such as the example of the hatching of the miracidia), it is not just the parasite's behavior that is affected by external variables, but also how the environment interacts with the parasite to develop different phenotypes. In this context, the concept of homeodynamics, discussed by Lloyd *et al*. [39] seems appropriate. It emphasizes “a capacity for bistable switching threshold phenomena”, that is, the system can transition between steady states and continuously transform its behavior without losing its overall structure. Whether the cell is the (sub)system, or the parasite, a dynamic system can respond to a stimulus in one of two ways – to either resist external influences, maintaining an internal state (e.g., remain a cercaria head after skin penetration or maintain the status of immature females after mating), or following a stimulus by changing the internal state (develop into a schistosomulum or develop into a mature female, respectively). In both cases, the temporary or permanent departure from a steady state is explained in terms of feedback mechanisms. The inheritance system would, therefore, receive a multitude of signals from the outside world but remain relatively stable until the ‘right’ stimulus arrives and triggers a switch in function. Therefore, ‘stabilizing’ factors must exist that maintain a current while ‘switching’ factors allow for choosing alternative trajectories at bifurcations. The art of experimental parasitology would consist of identifying these factors and all the elements of the inheritance system (such as the aforementioned epigenetic writers, readers, and erasers) that are receptive to these factors, as well as the threshold values for switching.

In addition, not all phenotypic variants that are produced will survive and, especially for larval forms, the environment will impose a strong selective pressure and reduce the number of phenotypes whose inheritance systems go from one developmental stage to the other and through subsequent generations.

## (*G*×*I*)×*E* in *S. mansoni* Ontogenesis: The Cercaria to Adult Transformation

Schistosomes undergo dramatic changes in their phenotype during their development from larvae into adults. Cercariae, for instance, are phenotypically completely different from adults. Naturally, the underlying expression pattern of genes and repetitive elements changes during this metamorphosis [40]. These transcriptional modifications are driven or accompanied by reorganization of the chromatin structure. One of the most surprising findings concerning these chromatin modifications was the discovery of a bivalent histone methylation on H3K4 and H3K27. H3K4me3 is a typical mark of active TSS while H3K27me3 is a repressive mark found in heterochromatin. Bivalent methylation is known to be a hallmark of a subset of genes in vertebrate embryonic stem cells and some cancer types (reviewed in [41]) where it holds transcription in a poised state allowing for rapid transcription upon exposure to external signals. The same is true for the cercarial genes with these modifications: these genes show very low levels of transcription, but become activated within a few hours when the H3K27 methylation is removed during the first developmental steps of the cercaria to schistosomulum transition [28].

Most of the parasitic flatworms are simultaneous hermaphrodites, in the sense that the individuals display both male and female reproductive organs. Schistosomatidae are an exception to this rule as they are gonochoric and have separate sexes (they are dioecious, that is, individuals are either male or female). The sex of schistosomes is genetically determined by ZZ (males) or ZW (females) sex chromosomes. There is, however, (i) no phenotypic dimorphism between males and females in the larval stages, and sexual dimorphism appears only in the vertebrate host, during schistosomula development; and (ii) there are apparently no female-specific genes. Accordingly, sexual differentiation does not rely solely on heritable factors (i.e., the sex chromosome) but also depends on environmental cues from the host as perceived differently by males and females 42, 43. Occasional cases of hermaphroditism in *S. mansoni* adult worms which developed in nonpermissive hosts further illustrate the importance of the host microenvironment for *Schistosoma* sexual differentiation 44, 45. Now, what role do epigenetic components play in this differentiation process? Sex-specific chromatin changes occur during sexual development from cercariae to adults and indicate an epigenetic component associated with the switch to sexual commitment [46]. From an evolutionary perspective, schistosome sex chromosomes display the characteristic features of sex chromosome evolution. Accumulation of repeats and heterochromatization of sex-determining regions occur before suppression of recombination between the heterochromosome and its homologue, likely restricting recombination of key loci involved in female fertility and male sterility, or vice versa [47]. Whether the accumulation of repeats is a cause or a consequence of heterochromatization remains an open question; however, other structural changes, such as chromosomal translocation or inversion, can also suppress recombination. In schistosomes, heterochromatization of the W chromosome has been known for a long time and was even used as a marker for sex identification in morphologically indistinguishable cercariae 48, 49. Further studies have shown that W-specific sequences are almost entirely composed of satellite-type repeats located in the heterochromatic region of the *Schistosoma* W chromosome. What makes *Schistosoma* sex chromosomes unique in comparison to other metazoan model species is that some of these W-specific repetitive DNA sequence elements are transcribed in the miracidial and cercarial stages but never in the adults, and this change in transcription level is accompanied by changes in the chromatin structure at these loci [22]. These repeats carry a euchromatic signature in miracidia and lose their euchromatic character progressively during the development into adults. In this sense, during the cercariae to schistosomula transition (when sexual dimorphism appears) the repeats heterochromatize. These findings led us to propose a scenario based both on genetic (W-specific repetitive elements) and epigenetic elements (reversible changes in chromatin structure) to explain the emergence of sex chromosomes in *Schistosoma* over evolutionary times [22]. In this model, transcription of satellite repeats leads in *cis* to heterochromatization of portions of the W-chromosome and/or in *trans* to chromatin structure changes on the Z-chromosome and/or the autosomes.

## (*G*×*I*)×*E* in Transgenerational Effects and Adaptive Evolution

Host–parasite interactions are characterized by strong mutualistic selective pressure. Parasites must find their hosts, adhere, penetrate, and survive not only a rapid change from external to internal environment but also the attack of the host's immune system. As digenean parasites, schistosomes must preserve in the definitive host the capacity to infect the intermediate snail host. One of the essential factors of infection success in *Biomphalaria glabrata* snails is a class of polymorphic glycoproteins called *S. mansoni* polymorphic proteins (*Sm*PoMuc). Their putative function and their interrelation with the snail immune response was recently exhaustively reviewed [50]. Several lines of evidence indicate that these proteins are important for penetration into the snail and/or for the very early developmental steps. Miracidia possess a large repertoire of *Sm*PoMucs despite having only about 10 *Sm*PoMuc genes. Transcriptional polymorphism is produced via strain-specific chromatin structure differences. By influencing this structure by pharmacological inhibition of histone-modifying enzymes (HME) or by strain hybridization, heritable *Sm*PoMuc expression polymorphism is generated that translates into an increase in snail infection rate 51, 52. A relative recent threat that the parasite faces is exposure to anthelminthic drugs. Despite this, drug resistance can emerge rapidly in natural populations [53] and can be generated within few generations in the laboratory [54]. Genetic mutations that confer the resistance to hycanthone and oxamniquine have been identified 55, 56, but hycanthone-resistant worms that do not carry the corresponding mutations were also found. These individuals showed a large panel of epiallelic changes compared to unexposed worms [57], indicating that hycanthone resistance or tolerance can also have an epigenetic basis. Today's most widely used antischistosomiasis drug is praziquantel (PZQ). Evidence for PZQ failure has been reported several times but there appears to be variability in the heritability of this trait when field isolates are used (reviewed in [58]). Interestingly, parasites that survive PZQ exposure do not show allele frequency distortions, suggesting that nongenetic inheritance could play a role in PZQ resistance [59].

## Concluding Remarks and Future Perspectives

When the G×E ⇒P concept was introduced originally, one of the immediate practical consequences was that (animal) breeding programs should be carried out in a range of different environments [11]. Equally, the major consequence of our systems approach to inheritance is that, if one wishes to understand the heritability of a trait, all elements of the inheritance system must be analysed comprehensively using a range of different genotypes and epigenotypes (and other elements of the inheritance system). This is, however, almost never feasible. To cope with the caveat, one should remember that the elements of the inheritance system are operationally defined and depend on the experimenter. It is legitimate to exclude (operationally) some of the elements from the experiment providing one does not exclude them from the conclusions and generalizations, for example, the finding that genetic variants have a strong association with a phenotype does not exclude similar or even stronger epiallelic associations and *vice versa*. Nevertheless, while the practical range of different genotypes and epigenotypes one can test will be low, it will be interesting to see how both alleles and epialleles are linked to phenotypes. In the simplest case, either epigenotype or genotype is kept invariant, and genetic or epigenetic loci responsible for the phenotypic trait are mapped using quantitative trait locus (QTL) or epiQTL, but it is conceivable that both approaches can be combined as long as epialleles and alleles are in different loci. Another consequence is that time matters: the process of the generation of the phenotype makes it implicit that it can only be understood over time. For the experimenter this means that correlations of genomes, epigenomes (or transcriptomes) with phenotypes are of limited value if one does not capture the dynamics of heritable and phenotypic changes. Instead of producing more replicates for one time point it might be more useful to produce time series with fewer replicates. Time matters also because the genotype and the epigenotype can have very different (epi)mutation and reversion rates 25, 60. Naturally, their relative importance in processes that are very long or very short will be different. It might, therefore, be useful to focus on epigenetic elements for short processes (ontogenic, and less than five meiotic generations) and on genetic elements for longer processes. Whatever strategy is used, the different elements of the inheritance system must be studied in an integrative and comprehensive approach (see Outstanding Questions) to better understand phenotypic variation and infection success of schistosomes.

Outstanding Questions What is the role of epigenetic participants such as DNA methylation, histones, nonhistone chromatin proteins, noncoding RNA, and nuclear topology in regulating *S. mansoni* gene expression and developmental biology?

Which are the chromatin signatures of each life stage of *S. mansoni*?

Which are the chromatin signatures of the recently described proliferating somatic cells of *S. mansoni*?

What new tools can be applied to study flatworm epigenetics?

How can a systems biology framework best be integrated with parasitology research?
