# Supplementary material for: (Epi)genetic Inheritance in Schistosoma mansoni: A Systems Approach
Source: Trends Parasitol. 2017 Apr;33(4):285–94. doi: 10.1016/j.pt.2016.12.002 (PMC6125318; doi:10.1016/j.pt.2016.12.002)
Supplement: Supplementary file 1 [file mmc1.pdf]

Question 1:

Schistosoma mansoni is ...

❍ A digenean hermaphroditic flatworm that causes river blindness

● A digenean gonochoric flatworm that causes intestinal bilharzia

Explanation:

Schistosomes are parasites whose life-cycle goes through two obligatory hosts (Di-genean). Each individual is either female of male. And they cause a parasitic disease that is called Schistosomiasis or Bilharzia to honor the doctor who discovered the worm in 1852, Theodor Bilharz. The adults of the species Schistosoma mansoni are usually found in the mesenteric veins. They produce eggs that penetrate the intestinal walls.

Reference:

Picard MA,et al: Sex-Biased Transcriptome of Schistosoma mansoni: Host-Parasite Interaction, Genetic Determinants and Epigenetic Regulators Are Associated with Sexual Differentiation. Plos NTD 2016, 10:e0004930.

❍ A digenean gonochoric cestode that causes intestinal bilharzia

❍ A digenean gonochoric flatworm that causes urinary bilharzia

Question 2:

A systems biology approach to inheritance

❍ represents a neo-Lamarckian alternative to the Modern evolutionary synthesis that underlines the importance of epigenetic inheritance in evolution

❍ is a concept in which inheritance is not only based on the genotype but on an inheritance system that is composed of several elements, such as genetic, epigenetic

but potentially also cytoplasmic, microbial or even cultural units that ar

- ● is a concept in which inheritance is not only based on the genotype but on an inheritance system that is composed of several elements, such as genetic, epigenetic but potentially also cytoplasmic, microbial or even cultural units that in

  Explanation:

  Exact. The units (or elements) are operationally defined, i.e. by the experimenter.

- ❍ allows to integrate data from genomics, transcriptomics, epigenomics, massive phenotyping etc. into sophisticated software solutions that allow for disentangling heritable and environmental effects on the phenotype

Question 3:

Epigenetic writers, readers and erasers are for instance ...

- ● histone methylases, chromo domain containing proteins, and histone demethylases

  Explanation:

  Histone modifying enzymes but also DNA (de)methylating enzymes are considered epigenetic readers and erasers because they add or remove epigenetic information to the DNA. These modifications can be recognized by proteins that bind to them (the readers) and that transform the epigenetic signals into gene function, e.g. inhibition or activation of transcription.

  Reference:

  Torres IO, Fujimori DG: Functional coupling between writers, erasers and readers of histone and DNA methylation. Curr Opin Struct Biol 2015, 35:68-75.

- ❍ scientists who write and read about epigenetics, and those (erasers) who don’t think that epigenetics is important. The role of epigenetics is controversial and after all science advances through fruitful discussions.

- ❍ large enzyme complexes that depose and remove (writers and readers) entire

nucleotides after replication (including epigenetic marks), and transcription factors in cooperation with RNApolII that can ‘erase’ epigenetic marks during transc

Question 4:

‘Chromatin colors’ are ...

❍ the original term of the dyes that were used to colorize the chromatin by Walther Flemming (1843-1905) who described the chromatin in 1882

❍ combinations of fluorescent dyes specifically designed to detect modified histones

❍ a simple way to provide consensus graphical output for the different histone modifications that is now used by all genome browsers (e.g. H3K9me3 = red, H3K9ac = green)

● combinations of epigenetic marks (e.g. histone modifications) that have functional importance for the organization of the chromatin

Explanation:

The idea to use ‘color’ as a signature for common combinations of different chromatin proteins that are present at specific sites was introduced by Filion et al. (2010) to "...avoid semantic confusion, and in line with the Greek word chroma (color)". In that work 5 common combinations of 53 chromatin proteins were labeled in the figures of the publication. The term was then used in several commentaries on this article and is now commonly employed to designate combinations of histone modifications.

Reference:

Filion GJ, van Bemmel JG, Braunschweig U, Talhout W, Kind J, Ward LD, Brugman W, de Castro IJ, Kerkhoven RM, Bussemaker HJ, van Steensel B: Systematic protein location mapping reveals five principal chromatin types in Drosophila cells. Cell 2010, 143:212-224.
